# Effect of Environmental Factors on Intra-Specific Inhibitory Activity of *Carnobacterium maltaromaticum*

**DOI:** 10.3390/microorganisms5030059

**Published:** 2017-09-14

**Authors:** Peipei Zhang, Mandeep Kaur, John P. Bowman, David A. Ratkowsky, Mark Tamplin

**Affiliations:** Tasmanian Institute of Agriculture, Food Safety Centre, University of Tasmania, Private Bag 54, Hobart, Tasmania 7001, Australia; mandeep.kaur@utas.edu.au (M.K.); john.bowman@utas.edu.au (J.P.B.); d.ratkowsky@utas.edu.au (D.A.R.); mark.tamplin@utas.edu.au (M.T.)

**Keywords:** *Carnobacterium maltaromaticum*, vacuum-packaged beef, intra-specific, bacterial interactions, inhibitory activity, pH, lactic acid, glucose, atmosphere, temperature

## Abstract

*Carnobacterium maltaromaticum* is frequently associated with foods having extended shelf-life due to its inhibitory activity to other bacteria. The quantification of such inhibition interactions affected by various environmental factors is limited. This study investigated the effect of environmental factors relevant to vacuum-packaged beef on inhibition between two model isolates of *C. maltaromaticum*, D0h and D8c, specifically D8c sensitivity to D0h inhibition and D0h inhibitor production. The effects of temperature (−1, 7, 15, 25 °C), atmosphere (aerobic and anaerobic), pH (5.5, 6, 6.5), lactic acid (0, 25, 50 mM) and glucose (0, 0.56, 5.55 mM) on D8c sensitivity (diameter of an inhibition zone) were measured. The effects of pH, glucose, lactic acid and atmosphere on D0h inhibitor production were measured at 25 °C. Sensitivity of D8c was the highest at 15 °C, under aerobic atmosphere, at higher concentrations of undissociated lactic acid and glucose, and at pH 5.5 (*p* < 0.001). pH significantly affected D0h inhibitor production (*p* < 0.001), which was the highest at pH 6.5. The effect of lactic acid depended upon pH level; at relatively low pH (5.5), lactic acid decreased the production rate (arbitrary inhibition unit (AU)/mL/h). This study provides a quantitative description of intra-species interactions, studied in in vitro environments that are relevant to vacuum-packaged beef.

## 1. Introduction

Bacteria interact in any given niche, and these interactions may have a positive or negative impact on the involved species [1]. Bacteria compete by secreting defensive compounds, directly ‘scrambling’ for nutrients or performing contact-dependent inhibition [1,2,3,4]. A type VI secretion delivery system (T6SS) is found to be utilized by *Pseudomonas aeruginosa* to deliver bacteriolytic effectors to other Gram-negative bacterial cells [5]. Furthermore, this tubular system has been identified in >80 Gram-negative bacterial genomes, including many members of the family *Enterobacteriaceae* [6]. Conversely, some bacteria are able to cooperate by exchanging metabolic products or via quorum sensing systems [1,7,8,9]. For example, *Bifidobacterium bifidum*, a probiotic organism that is often found in human intestines, and *Listeria monocytogenes*, an opportunistic pathogen, mutually promote each other’s growth [10]. According to D'Onofrio et al. [11], previously uncultured bacteria from marine sediment grew on agar in the presence of readily-cultured bacteria due to the latter forming growth-promoting siderophores.

Bacterial interactions have been frequently reported in food-relevant environments. A few researchers have observed the inhibitory effect of natural microbiota of meat on the growth of pathogens, which has been defined as the ‘Jameson effect’ [12,13,14]. Nychas et al. [15] found that the growth rates of both *Serratia marcescens* and *Pseudomonas fluorescens*, the main spoilage organisms in pork, were increased by quorum sensing compounds in vitro. A large number of studies regarding interactions among food-sourced bacterial species mainly focus on the inhibitory activity of lactic acid bacteria (LAB) on pathogenic or spoilage bacteria in vitro or in situ within food [16,17,18]. Such interactions are important in influencing the microbial community structure, yet we have a very limited understanding about the quantitative nature of microbial interfaces, including the influence of the environment [19,20,21]. The information on the strength of bacterial interactions may help us to better utilize these interactions and further to control food safety, quality and food waste, which is particularly relevant for nutritious and perishable products such as meat.

Among the numerous microorganisms that contaminate food, *Carnobacterium maltaromaticum* is a dominant, and often preferred, LAB species. Numerous reports demonstrate that its presence is associated with foods having an extended shelf-life, including vacuum-packaged (VP) meat [16,22,23,24]. For example, in climax microbial communities, *C. maltaromaticum* may be represented by a single strain, and the effect is likely attributed to bacteriocins—inhibitory compounds that suppress other bacteria within the spoilage community [23].

Examining a broad spectrum of bacterial species isolated from VP beef produced at different abattoirs, we showed that cell-free supernatant (CFS) of *C. maltaromaticum* (e.g., strain D0h) inhibited a wide spectrum of species, including *Serratia* spp., *Pseudomonas* spp., *Leuconostoc* spp. and other *Carnobacterium* spp. [25]. Under aerobic conditions, most *Pseudomonas* spp. and two *Bacillus* spp. showed relatively large inhibitory activities. Interestingly, the CFS of a few Gram-negative bacteria including *Pseudomonas* spp. and *Enterobacteriaceae* also showed growth-promoting activity. That study has helped to understand bacterial interactions among a large variety of species related to VP beef. However, a more complete understanding of how populations of species and strains interact and compete requires a quantitative description of the effect(s) of environmental factors.

The present study describes intra-specific inhibitory activity between two model VP beef bacterial isolates, *C. maltaromaticum* D0h and D8c, specifically the effects of environmental factors relevant to VP beef. Firstly, a preliminary test was performed to investigate the production kinetics of inhibitory compounds by D0h, and then, D0h CFS with the highest level of inhibition was obtained to study the effects of pH, atmosphere, glucose, temperature and lactic acid on D8c sensitivity under an agar model system with the diameter of an inhibition zone measured. To better understand the inhibition between *C. maltaromaticum* D0h and D8c, the production of D0h inhibition in broth medium with various combinations of pH, atmosphere, glucose and lactic acid was also tested at 25 °C.

## 2. Materials and Methods

### 2.1. Bacterial Isolates

Effector and target strains of *C. maltaromaticum*, D0h and D8c, respectively, were isolated from VP beef [26,27] and stored at −80 °C. Isolates were cultured on tryptone soy agar (TSA, Oxoid Ltd., Thebarton, Australia) and then incubated separately in brain heart infusion broth (BHI, Amyl Media Ltd., Adelaide, Australia) at 25 °C for 24 h prior to experiments.

### 2.2. Production Kinetics of Inhibitory Compounds by C. maltaromaticum D0h

*Carnobacterium maltaromaticum* D0h was inoculated in BHI at an initial cell density of 10^5^ CFU/mL and then incubated at 25 °C. Broth was sampled every 3 h for the first 9 h and then at 1.5-h intervals, and the optical density was measured at 600 nm (OD_600_) by a plate reader (SPECTROstar Nano Absorbance Reader, Ortenberg, Germany). D0h CFS was prepared by centrifuging cultures at 10,000× *g* for 5 min, followed by filtration through a 0.2 µm pore-sized membrane (Corning^®^, Berlin, Germany). The inhibitory activity of CFS was determined by an agar overlay assay reported by Aween et al. [28], with slight modification. Briefly, 24-h culture (25 °C) of D8c (indicator/target isolate) was adjusted to 10^8^ CFU/mL (OD_600_, 0.10–0.15), and then 10 mL of melted TSA (0.7% agar, g/v; 50 °C) were mixed with 10^7^ CFU D8c and poured into Petri dishes. After the agar had solidified, 10 µL of filter-sterilized CFS of D0h were spotted on the agar in triplicate using a micropipette. After incubation at 25 °C for 24 h, the diameter (mm) of inhibition (DI) was measured using ImageJ software (v1.49 (http://rsb.info.nih.gov/ij/index.html)).

### 2.3. Effect of Environmental Factors on the Sensitivity of C. maltaromaticum D8c to C. maltaromaticum D0h CFS

The sensitivity of the target isolate D8c to inhibitory activity of D0h CFS was tested under environmental conditions relevant to VP beef: temperature: −1, 7, 15 and 25 °C; atmosphere: aerobic and anaerobic; pH: 5.5, 6 and 6.5; lactic acid: 0, 25 and 50 mM; glucose: 0, 0.56 and 5.55 mM. A full factorial design (4 × 2 × 3 × 3 × 3) was used.

The agar overlay method was used with a basal medium of modified brain heart infusion (mBHI) broth, without glucose (AM 11-NG, Amyl Media, Ltd., Dandenong, Australia; mBHI contained 10 g blended peptone No. 1, 5 g sodium chloride, 17.5 g brain heart infusion solid and 2.5 g di-sodium hydrogen orthophosphate, per litre). L (+) lactic acid (Scharlab, Barcelona, Spain) and D (+) glucose (Sigma, St. Louis, MO, USA) were added into 27 variations of mBHI (Table 1). pH was adjusted to 5.5, 6 or 6.5 (±0.1) using 10 M NaOH or 10 M HCl (Table 1). Agar was added, the medium autoclaved and then the pH adjusted. To better understand the mechanism of the effect of lactic acid, undissociated lactic acid (UndisLA) was calculated as $\left[\mathrm{UndisLA}\right]=\frac{\left[\mathrm{LA}\right]}{1+\;10^{\left(\mathrm{pH}-3.86\right)}}$.

After cooling to 50 °C, 10 mL mBHI agar were mixed with 10^7^ CFU D8c and added to Petri dishes; the dishes were previously prepared with a bottom layer containing 15 mL of the same mBHI agar used in the overlay.

**Figure 1:**
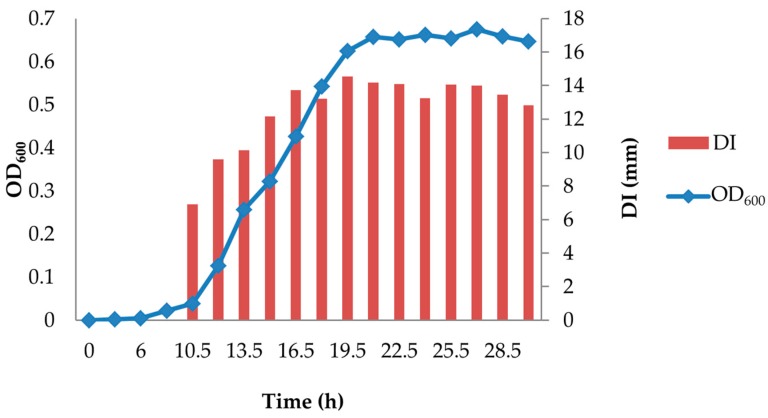
Kinetics of inhibitory compound production by *C. maltaromaticum* D0h. DI, diameter of inhibition zone.

Results showed that the highest levels of inhibitory compounds (DI, ~14 mm) in D0h broth cultures occurred between 19.5 and 22.5 h of incubation (Figure 1). Therefore, the CFS was harvested during this time interval, and three 10 µL aliquots were added to the agar surface as replicates for each agar plate. Three agar plates were used as triplicates for each combination of environmental factors.

After the CFS was absorbed into agar, Petri dishes were incubated at different temperatures both aerobically and anaerobically until inhibition zones were observed (Table 1 and Table 2).

Anaerobic conditions (<1.0% O_2_, ≥13% CO_2_) were produced using a GasPak EZ anaerobic pouch system (BD, Blackburn, Australia) in sealed jars. Incubation temperature was recorded during the experiment with data loggers (Thermochron iButton^®^, Warrnambool, Australia). At the end of the experiments, DI was measured and defined as D8c sensitivity.

### 2.4. Effect of PH, Lactic Acid, Glucose and Atmosphere on Inhibition Level of D0h CFS

A full factorial design (2 × 3 × 3 × 3) of atmosphere, lactic acid, glucose and pH was used in 54 combinations of the four environmental factors. Using BHI (without glucose) as the basal medium, 27 formulations of modified BHI broth medium (mBHI) were prepared, based on the factorial design (Table 1). 

The 1.8 mL of mBHI broth were added to individual wells of a 24-well plate and then inoculated with 200 µL of *C. maltaromaticum* D0h 24-h culture, for an initial density of 10^5^ CFU/mL. Two replicate wells were used for each test condition. Plates were incubated at 25 °C, aerobically and anaerobically, respectively. When culture OD_600_ reached 0.05, samples were collected at 2-h intervals until the bacteria reached the stationary phase. CFS was obtained by filtering the two replicate samples through 0.2-µm filters and serially diluted using BHI.

The level of inhibition in CFS was measured by the agar overlay method described above, with modification to increase detection sensitivity. Instead of TSA, agar medium (pH 6.5 ± 0.1) was made with 37 g/L BHI, 5.06 g/L L(+)-lactic acid and 15 g/L agar. Furthermore, the cell density of target bacteria, *C. maltaromaticum* D8c, was reduced to 10^4^ CFU. A mixture of molten medium and target bacteria was poured over the solidified agar made from the same medium. After air-drying in a hood, 10 µL of each CFS dilution were spotted onto the solidified agar surface and incubated at 25 °C for 48 h. The highest dilution of CFS showing inhibition was defined as one arbitrary inhibition unit (AU) [29]. Each combination of the 54 conditions was performed twice. The relationship between sampling time (h) and concentration of inhibitory compounds (AU/mL) was calculated and the production rate (AU/mL/h) determined by linear regression in Excel^®^ (v2010; Microsoft Corp., Redmond, WA, USA).

The inhibitor production at biomass level was also determined. Firstly, the bacterial cell density (log_10_ CFU/mL) of each sample was calculated according to a regression equation between cell density and log OD_600_: cell density = optical density*1.1168 + 9.4726 (R^2^ = 0.996), which was developed in a preliminary experiment (Figure S1). A linear regression between the concentration of inhibitory compounds and culture cell density was then determined in Excel^®^. The production of inhibitory compounds by D0h per log CFU was calculated from the slope of the regression line and designated as logAU/logCFU.

### 2.5. Data Analysis

The overall effect of environmental factors on D8c sensitivity and D0h inhibitor production (per hour and per log CFU) was evaluated using analysis of variance (ANOVA), employing the GLM (general linear model) procedure in SAS (v9.3; SAS, Inc., Rockville, MD, USA). If the *p*-value from the F-test was below 0.05, a Student *t*-test was then applied to identify significant (*p* < 0.05) pairwise differences. The correlation coefficient between undissociated lactic acid and DI and undissociated lactic acid and inhibitor production (AU/mL/h) was calculated via linear regression in Excel^®^.

## 3. Results

### 3.1. Kinetics of D0h Inhibitory Activity Production

Inhibitory activity (DI) of D0h CFS was detected between 6 and 9 h of incubation with a blurred inhibition zone; hence, DI was not determined for this time point (Figure 1). Inhibitory activity increased dramatically during the exponential phase of bacterial growth and then reached a maximum level (DI, 14.5 mm; 19.5 h) corresponding to the early stationary phase.

### 3.2. Environmental Effects on D0h Inhibitor Production

**Figure 2:**
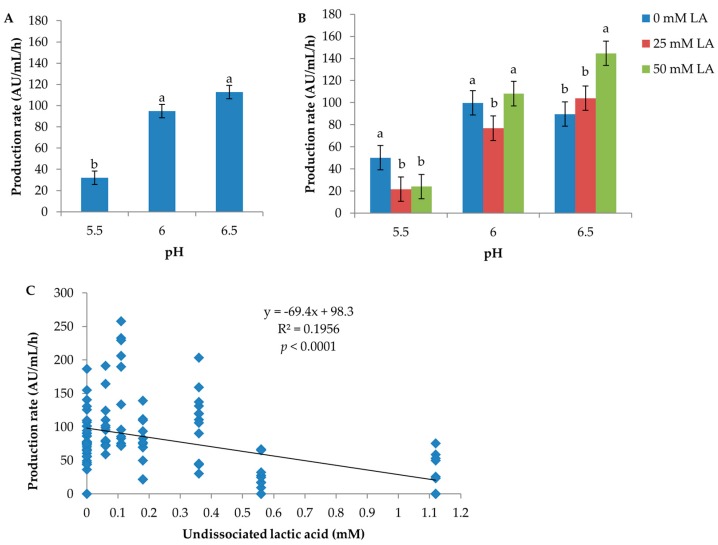
Effect of pH and lactic acid on inhibitor production (AU/mL/h) by *C. maltaromaticum* D0h. In (**A**,**B**), the error bar represents the standard error of the mean; means with the same letters are not significantly different. In (**B**), means having the same level of pH, but differing amounts of total lactic acid were compared. (**C**) shows the production rates achieved from all culture conditions and grouped according to the calculated amount of undissociated lactic acid.

pH had a significant effect both on inhibitor production per hour and per biomass unit (CFU) (Table S1). The rate of inhibitor production per hour was inversely related to pH; pH 6.5: 112.8 AU/mL/h; pH 6: 94.9 AU/mL/h; pH 5.5: 32.0 AU/mL/h (Figure 2A). The inhibitor production per biomass unit was lowest at pH 5.5 (0.8 log AU/log CFU) and ≥1.2 logAU/logCFU at both pH 6 and 6.5 (Figure S2). Inhibitory activity was not detected in media containing 50 mM lactic acid at pH 5.5 under anaerobic conditions (Medium No. 7, 16 and 25 in Table S2) and at pH 5.5 in medium containing 25 mM lactic acid and 5.55 mM glucose incubated aerobically and anaerobically (Medium No. 22 in Table S2). 

The effect of lactic acid on production rate (AU/mL/h) was heavily dependent on the pH level of a culture condition, with *p* = 0.0098 for the interaction of lactic acid with pH (Table S1). At pH 5.5, the production rate was the greatest with no lactic acid supplementation (50.2 AU/mL/h), followed by 25 and 50 mM lactic acid (21.7 and 24 AU/mL/h, respectively) (Figure 2B). Conversely, at pH 6.5, the production rate was the highest (144.6 AU/mL/h) in the presence of 50 mM lactic acid. At pH 6, lactic acid showed a rather different effect pattern compared to pH 5.5 and 6.5; the mean production rate produced in media containing 25 mM LA was statistically significantly lower than that produced in media containing 0 and 50 mM lactic acid according to the standard error of the mean calculated by ANOVA of pooled data. However, a review of the original data found that the experimental variation at pH 6 was large. For example, one replicate produced very low production rates in Medium No. 5 aerobically (21.74 AU/mL/h) and anaerobically (22.13 AU/mL/h), respectively, compared to the other replicate (110.14 and 76.88 AU/mL/h, respectively) (Table S2). Hence, we would conclude that the significance of the difference of mean production rate between pH 6 and pH 5.5 and 6.5 was false. The production rate as a function of the level of undissociated lactic acid is plotted in Figure 2C; the production rate decreases dramatically when the concentration of undissociated lactic acid reaches 0.56 mM and above. No effect of lactic acid on production per biomass unit (CFU) was observed (*p* = 0.588, Table S1).

The overall effect of glucose on production rate (AU/mL/h) was not significant (*p* = 0.404) (Table S1). At pH 6, the production rates in media with three glucose levels were not different; at pH 6.5, it fluctuated in a haphazard way; at pH 5.5, the mean production rate was the lowest (11.2 AU/mL/h) in the media with 5.55 mM glucose, which was likely the reason for the statistically-significant interaction of glucose with pH (*p* = 0.0017) (Figure S3). Further analysis of the data found that the lowest mean production rate at pH 5.5 was due to D0h failing to produce inhibitory compounds at a few culture conditions (anaerobic, Medium No. 19, 22 and 25; aerobic, Medium No. 22) (Table S2), which also explained the relatively overall lower mean production per CFU in media with 5.55 mM glucose (Figure S2C, Table S1). Hence, it was concluded that glucose did not have an effect on either the production per hour or per biomass unit.

In addition, inhibitor production was not significantly affected by atmosphere (*p* = 0.236 for production per hour and *p* = 0.731 for production per CFU, respectively) (Table S1), the production rates in aerobic (84.3 AU/mL/h) and anaerobic (75.5 AU/mL/h) atmospheres not being significantly different (Figure S3A).

### 3.3. Influence of Environmental Factors on D8c Sensitivity to D0h CFS

**Figure 3:**
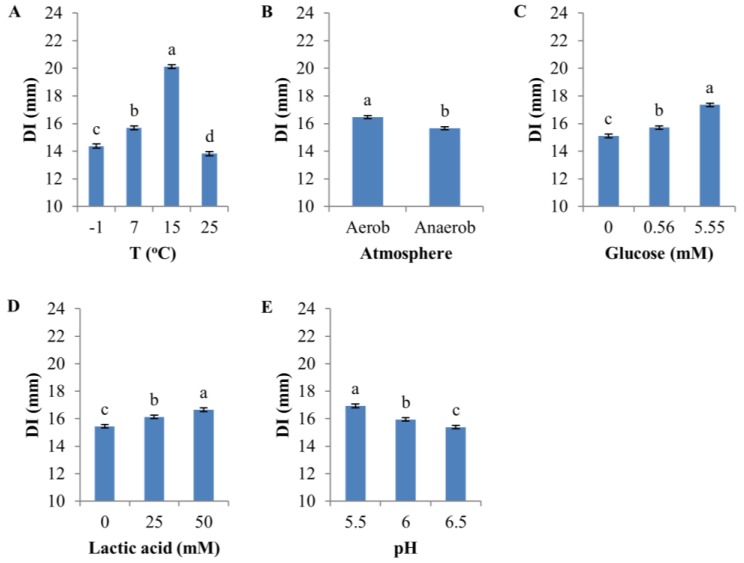
Effect of environmental factors on the diameter of inhibition zone (DI). (**A**) Temperature; (**B**) Atmosphere (aerobic vs. anaerobic); (**C**) Glucose; (**D**) Lactic acid; (**E**) pH. The error bar represents the standard error of the mean. Means with the same letter in each panel are not significantly different.

All environmental factors had a significant effect (*p* < 0.0001, F-test) on the sensitivity of D8c to D0h CFS (Figure 3). The highest temperature (25 °C) produced the lowest sensitivity (mean, 13.8 mm), whereas the greatest sensitivity (20.1 mm) occurred at 15 °C, followed by 7 and −1 °C (Figure 3A and Figure S4).

Overall, aerobic atmosphere only slightly increased sensitivity (mean DI = 16.5 mm) compared to anaerobic conditions (mean DI = 15.7 mm) (Figure 3B and Figure S5, and Table S3), but the difference was highly significant statistically (*p* < 0.0001, Table S3). Glucose and lactic acid both significantly increased DI (Figure 3C,D, Figures S6 and S7); on average, DI was 2.3 mm greater at 5.55 mM compared to no glucose supplementation, and lactic acid increased D8c sensitivity by 1.2 mm in the presence of 50 mM lactic acid compared with no lactic acid supplementation. Sensitivity was inversely related to pH (mean of DI, pH 6.5: 15.4 mm; pH 6.0: 15.9 mm; pH 5.5: 16.9 mm) (Figure 3E and Figure S8).

### 3.4. Interactions among Environmental Factors on D8c Sensitivity

**Figure 4:**
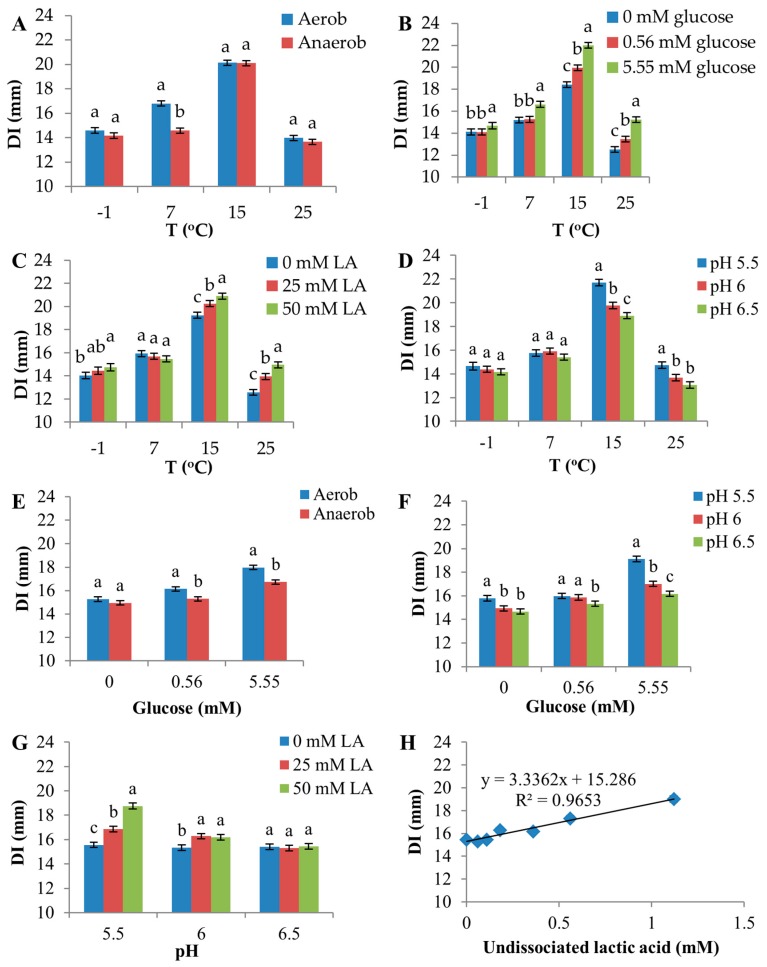
Effects of interactions among environmental factors on the diameter of inhibition zone (DI). In (**A,E**), Aerob signifies aerobic atmosphere; Anaerob signifies anaerobic atmosphere. The two-factor interactions shown in (**A**–**D**,**F**,**G**) were significant (F-test, *p* < 0.05). The interaction shown in (**E**) was not statistically significant (*p* = 0.055). (**H**) shows a linear regression between DI and undissociated lactic acid. Error bars are the standard error of the mean. Means within the same level of the factor shown on the x-axis were compared, and different letters indicate significant differences.

Significant interactions were observed between temperature and atmosphere, temperature and glucose, temperature and lactic acid, temperature and pH, glucose and pH and pH and lactic acid (Figure 4, Table S3). A slight interaction between glucose and atmosphere was observed, although it was not statistically significant (*p* = 0.0545).

Atmosphere produced a strong discriminating effect on D8c sensitivity at 7 °C, compared with other temperatures (Figure 4A), as the aerobic environment increased DI by 2.2 mm compared with the anaerobic environment at this temperature. In contrast, higher temperatures increased the inhibitory effect of glucose, lactic acid and low pH (Figure 4B–D). For example, at 15 and 25 °C, 5.55 mM glucose increased DI by 3.6 and 2.7 mm, respectively, compared with 0 mM glucose (Figure 4B). However, at −1 °C, DI increased by only 0.6 mm for this change in glucose.

The effects of atmosphere and pH on D8c sensitivity were greater in the presence of 5.55 mM glucose (Figure 4E,F, respectively). Furthermore, as a result of a relatively larger portion of the undissociated form of lactic acid added in media with low initial pH compared with high initial pH, 50 mM lactic acid increased DI by 3.2 mm compared to 0 mM lactic acid at pH 5.5, whereas at pH 6.0, the increase was only 0.9 mm, and at pH = 6.5, there was no measurable increase (Figure 4G). These responses are a consequence of the positive correlation between undissociated lactic acid and DI (R^2^ = 0.965) (Figure 4H).

## 4. Discussion

Bacterial interactions have long been investigated in culture media and food model systems. For example, *Brochothrix thermosphacta* was inhibited by the presence of LAB in agar media [30]. The presence of *Pseudomonas* spp. was found to enhance the survival of the pathogenic species *Campylobacter jejuni* in vitro and in poultry [31,32]. In this study, by using an agar model system, we have quantified the effects of the VP beef-related environmental factors, pH, lactic acid, glucose, temperature and atmosphere on the inhibitions between two *C. maltaromaticum* isolates, i.e., D8c sensitivity and D0h inhibitor production.

The pH of fresh VP beef ranges from approximately pH 5.5–6.5 [33,34,35]. *C. maltaromaticum* D8C sensitivity to D0h CFS, interpreted by DI, increased at lower pH, agreeing with Ganzle et al. [36], who reported that the inhibitory activity of nisin, sakacin P and curvacin A increased at low pH in broth media. Abriouel et al. [37] proposed that H^+^ affected bacteriocin activity via changes in the surface charge of target bacteria, thereby causing changes in conformation/oligomerization of bacteriocin peptides. Nisin is believed to have greater activity in acidic foods, due to increased solubility and stability [38]. It is also possible that a larger proportion of organic acids produced by *C. maltaromaticum* D0h is undissociated at low pH (5.5), which increased the overall inhibitory activity of D0h CFS. We found that D8c cell density in agar was reduced at lower pH (Figure S9A) and that D8c growth rate in mBHI broth (25 °C) decreased proportionally with lower pH (Figure S10). Therefore, larger inhibition zones at lower pH may have been influenced by a higher ratio of inhibitor per cell.

Lactic acid, well-known to inhibit the growth of pathogenic and spoilage bacteria and frequently applied as a food preservative [39,40,41], potentiated the sensitivity of D8c to D0h CFS. This effect is likely owing to the undissociated form of lactic acid, which has strong inhibitory activity due to lipophilic properties, enabling it to diffuse through bacterial membranes [39,42,43,44]. A significant interaction between lactic acid and pH on DI was observed, with lactic acid producing a greater effect at lower pH (Figure 4G) and showing a positive linear relationship between DI and undissociated lactic acid (Figure 4H). Hence, the effect of lactic acid likely results from the undissociated form. As with lower pH, greater levels of lactic acid in mBHI agar resulted in lower final D8c cell density (Figure S9B), indicating that DI could be influenced by a higher ratio of inhibitor compound per target cell.

Inhibitor activity was greater in the presence of 5.55 mM compared with 0.56 mM glucose and when mBHI agar was not supplemented with glucose (Figure 3C). *C. maltaromaticum* utilizes glucose and produces organic acids [45,46], resulting in 0.21 mole of L-lactic acid per mole of glucose [47]. To investigate the mechanism of the glucose effect on DI, pH change during incubation was measured for mBHI agar incubated at 15 °C. Average pH decreased 0.4 units in 5.55 mM glucose, which was greater than the pH decrease for 0 and 0.56 mM glucose, which were 0.09 and 0.1, respectively. Therefore, the enhancing effect of glucose on intra-specific inhibitory activity might be due to increased lactic acid, which decreased pH during D8c growth via glucose metabolism. This idea is consistent with interactions between glucose and pH (Figure 4F); with 5.55 mM glucose, D8c was more sensitive to pH change compared to 0 and 0.56 mM glucose. As with pH and lactic acid, final cell density was lower in mBHI agar containing higher glucose levels (Figure S9C). Other studies have shown that the inhibitory activity of two-component bacteriocins, such as lactocin 705 and lacticin 3147, is enhanced when target cells are energized due to the uptake of glucose [48,49].

Inhibition by D0h CFS was greater under aerobic versus anaerobic conditions (Figure 3B). According to Afzal et al. [46], glucose metabolism by *C. maltaromaticum* LMA28 is higher in the presence of oxygen, and consequently, the production of lactic acid increases under aerobic conditions. This is likely the case in our study, where enhanced antibacterial activity of D0h CFS may result from increased production of lactic acid. This may also explain the interaction observed between atmosphere and glucose, although it was only marginally significant (*p* = 0.055) (Figure 4E), with the difference of DI between aerobic and anaerobic conditions being greatest at 5.55 mM glucose.

Due to lower growth rates at −1, 7 and 15 °C, mBHI agar was incubated for longer time periods (Table 2) compared to 25 °C. Preliminary experiments demonstrated that DI did not increase with increased incubation time (Figure S11). Interestingly, inhibitory activity did not linearly correlate with temperature, where DI was higher at 15 °C, compared to −1, 7 and 25 °C (Figure 3A). We suggest that the sensitivity of D8c to D0h inhibition (DI) is dependent on the physiological state of D8c target cells, which may be influenced by temperature [50]. Bacteriocins are known to interact with the cytoplasmic membrane of sensitive bacteria [3,38,51,52,53], and Jacquet et al. [50] reported that the effect of class IIa bacteriocins depended on the physiological state of target bacteria. Henry et al. [54] reported that the lethal effect of carnocin CP5 was lower, but more prolonged, in the range of 7–30 °C; however, Stoffels et al. [55] found that a bacteriocin produced by *C. maltaromaticum* had no effect at 4 and 15 °C. 

To understand inhibition between *C. maltaromaticum* D0h and D8c better, the effects of pH, glucose, atmosphere and lactic acid on inhibitor production by D0h were investigated at 25 °C. *C. maltaromaticum* D0h produced small or no detectable levels of inhibitory compounds in media with initial pH 5.5. This may result from a bacterial strategy to shift energy from biosynthesizing metabolites and translocating them to the external medium, to maintaining internal pH in high H^+^ environments [56,57]. Relatively high pH has been shown to be optimal for the production of bacteriocins by *C. maltaromaticum* [56,58]. As mentioned earlier, undissociated lactic acid can inhibit bacterial growth [44,59]; we found that CFS inhibitor production rate decreased when the concentration of undissociated lactic acid was above 0.56 mM (Figure 2C).

In our study, the inhibitory effect produced by D0h and the sensitivity of D8c to inhibition were studied separately. The effects of environmental factors were complicated, not only affecting inhibitor production, but also sensitivity and/or growth of D8c. Nevertheless, it is possible to estimate the effects of environmental factors on the net outcome of the bacterial interaction under a few conditions. For example, at 25 °C and pH 6, glucose increased D8c sensitivity, but did not significantly affect D0h inhibitor production (Figures S3D and S12A); hence, it is likely the overall D0h-D8c inhibition strength is higher in culture conditions with a relatively higher concentration of glucose. However, at pH 5.5, lactic acid increased D8c sensitivity (Figure S12B), but decreased D0h production rate (Figure 2B). In this instance, it is more difficult to predict the absolute additive effect of these factors on inhibition strength. Similarly, relatively low pH increased D8c sensitivity (Figure 4D), while decreasing the inhibitor production rate by D0h (Figure 2A), making it difficult to evaluate the overall effect of pH on inhibition strength between the *C. maltaromaticum* strains. Under aerobic conditions, the D8c sensitivity and D0h inhibitor production rate were both slightly higher (although not statistically significant) compared to an anaerobic environment (Figure 4A and Figure S3A). Therefore, D0h-D8c inhibition strength would likely be enhanced in the presence of O_2_. It is also possible that residual O_2_, especially at the beginning of the storage of VP beef, may benefit inhibitor strains of *C. maltaromaticum* to compete against sensitive strains.

This study focused on the effects of environmental conditions on the intra-specific inhibitory activity between two model bacteria, *C. maltaromaticum* strains, D0h vs. D8c. Experimental data were generated in vitro to define the effects of environmental factors more clearly, without the potential complicating factors of a complex meat matrix. These findings are an important first step to help to explain future research that will focus on monitoring the growth of a microbial community including different bacterial strains and/or species. Such bacteriological medium-based studies have been extensively used to understand how environments influence bacterial growth, resulting in predictive models [60,61,62,63], which are subsequently validated in food matrices.

## Figures and Tables

**Table 1:** Levels of lactic acid, glucose and pH for modified brain heart infusion (mBHI) formulations.

Medium (No.)	Lactic Acid (mM)	Glucose (mM)	pH	UndisLA (mM) ^1^
1	0	0	5.5	0
2	0	0	6	0
3	0	0	6.5	0
4	25	0	5.5	0.56
5	25	0	6	0.18
6	25	0	6.5	0.06
7	50	0	5.5	1.12
8	50	0	6	0.36
9	50	0	6.5	0.11
10	0	0.56	5.5	0
11	0	0.56	6	0
12	0	0.56	6.5	0
13	25	0.56	5.5	0.56
14	25	0.56	6	0.18
15	25	0.56	6.5	0.06
16	50	0.56	5.5	1.12
17	50	0.56	6	0.36
18	50	0.56	6.5	0.11
19	0	5.55	5.5	0
20	0	5.55	6	0
21	0	5.55	6.5	0
22	25	5.55	5.5	0.56
23	25	5.55	6	0.18
24	25	5.55	6.5	0.06
25	50	5.55	5.5	1.12
26	50	5.55	6	0.36
27	50	5.55	6.5	0.11

^1^ UndisLA, the undissociated form of lactic acid (Materials and Methods, Section 2.3).

**Table 2:** Incubation conditions for DI measurement.

Temperature (°C)	Atmosphere		Medium No. ^1^	Incubation Time (Days)
	Aerobic	Anaerobic		
25	+	+	1–27	10
15	+	+	1–27	19
7	+	+	1–27	45
−1	+	+	1–3, 5, 6, 8–12, 14, 15, 17–21, 23, 24, 26, 27	145
−1	+	+	13	263
−1		+	4, 16, 22	263
−1	+	+	7, 25	NG ^2^
−1	+		4, 16, 22	NG

^1^ Medium No. is described in Table 1. ^2^ NG, no visible growth of target bacterium was observed; diameter of inhibition zone not measured.

## Supplementary Materials

The following are available online at www.mdpi.com/2076-2607/5/3/59/s1, Figure S1: Linear regression between cell density and optical density of *C. maltaromaticum* D0h in culture medium, Figure S2: Effect of environmental factors on the production of inhibitory factor by *C. maltaromaticum* D0h per CFU, Figure S3: Effect of atmosphere, glucose, lactic acid and glucose*pH on *C. maltaromaticum* D0h inhibitor production (AU/mL/h), Figure S4: Representative pictures of the inhibition zone showing the effect of temperature on D8c sensitivity (diameter of inhibition zone), Figure S5: Representative pictures of the inhibition zone showing the effect of atmosphere on D8c sensitivity (diameter of inhibition zone), Figure S6: Representative pictures of the inhibition zone showing the effect of glucose on D8c sensitivity (diameter of inhibition zone), Figure S7: Representative pictures of the inhibition zone showing the effect of lactic acid on D8c sensitivity (diameter of inhibition zone), Figure S8: Representative pictures of the inhibition zone showing the effect of pH on D8c sensitivity (diameter of inhibition zone), Figure S9: Effect of pH, lactic acid and glucose on the final cell density (FCD) in the study of *C. maltaromaticum* D8c sensitivity, Figure S10: Effect of pH on the growth rate of *C. maltaromaticum* D8c, Figure S11: Effect of incubation time on the diameter of inhibition zone (DI) at different incubation days, Figure S12: Effect of interactions between pH and glucose and pH and lactic acid on the diameter of the inhibition zone (DI) at 25 °C, Table S1: Significance (*p*-value, F-test) of the effect of environmental factors on the production of inhibitory compounds by *C. maltaromaticum* D0h, Table S2: D0h inhibitor production in various culture conditions, Table S3: Significance (*p*-value, F-test) of the effect of environmental factors on *C. maltaromaticum* D8c sensitivity to *C. maltaromaticum* D0h CFS, Table S4: The sensitivity of *C. maltaromaticum* (diameter of inhibition zone (DI)) D8c to *C. maltaromaticum* D0h inhibition under aerobic conditions, Table S5: The sensitivity of *C. maltaromaticum* (diameter of inhibition zone (DI)) D8c to *C. maltaromaticum* D0h inhibition under anaerobic conditions, Table S6: Significance (*p*-value, F-test) of the effect of environmental factors on *C. maltaromaticum* D8c sensitivity to *C. maltaromaticum* D0h CFS at 25 °C.
