# Supplementary material for: Effect of Environmental Factors on Intra-Specific Inhibitory Activity of Carnobacterium maltaromaticum
Source: Microorganisms. 2017 Sep 14;5(3):59. doi: 10.3390/microorganisms5030059 (PMC5620650; doi:10.3390/microorganisms5030059)
Supplement: Supplementary file 1 [file microorganisms-05-00059-s001.zip › Supplementary file-Figures.docx]

Supplementary Materials: Effect of Environmental Factors on Intra-Specific Inhibitory Activity of *Carnobacterium altaromaticum*

Peipei Zhang, Mandeep Kaur, John P. Bowman, David A. Ratkowsky and Mark Tamplin

**Figure S1.** Linear regression between cell density and optical density of *C. maltaromaticum* D0h in culture medium.

Overnight (24 h) cultures of *C. maltaromaticum* D0h were diluted in two–fold serial dilutions, using BHI. For each dilution, OD_600_ was measured and cell density determined by plate count. The linear regression between the two variables was calculated in Excel ® (v2010; Microsoft Crop).

**Figure S2.** Effect of environmental factors on production of inhibitory factor by *C. maltaromaticum* D0h per CFU.

*P*–value of F–test for panel A, B, C, and D is 0.003, 0.588, 0.049 and 0.731, respectively. Error bar represents standard error of mean. Means with the same letters are not significantly different.

**Figure S3.** Effect of atmosphere, glucose, lactic acid and glucose*pH on *C. maltaromaticum* D0h inhibitor production (AU/mL/h).

Error bar represents standard error of mean. Means with the same letters are not significantly different.


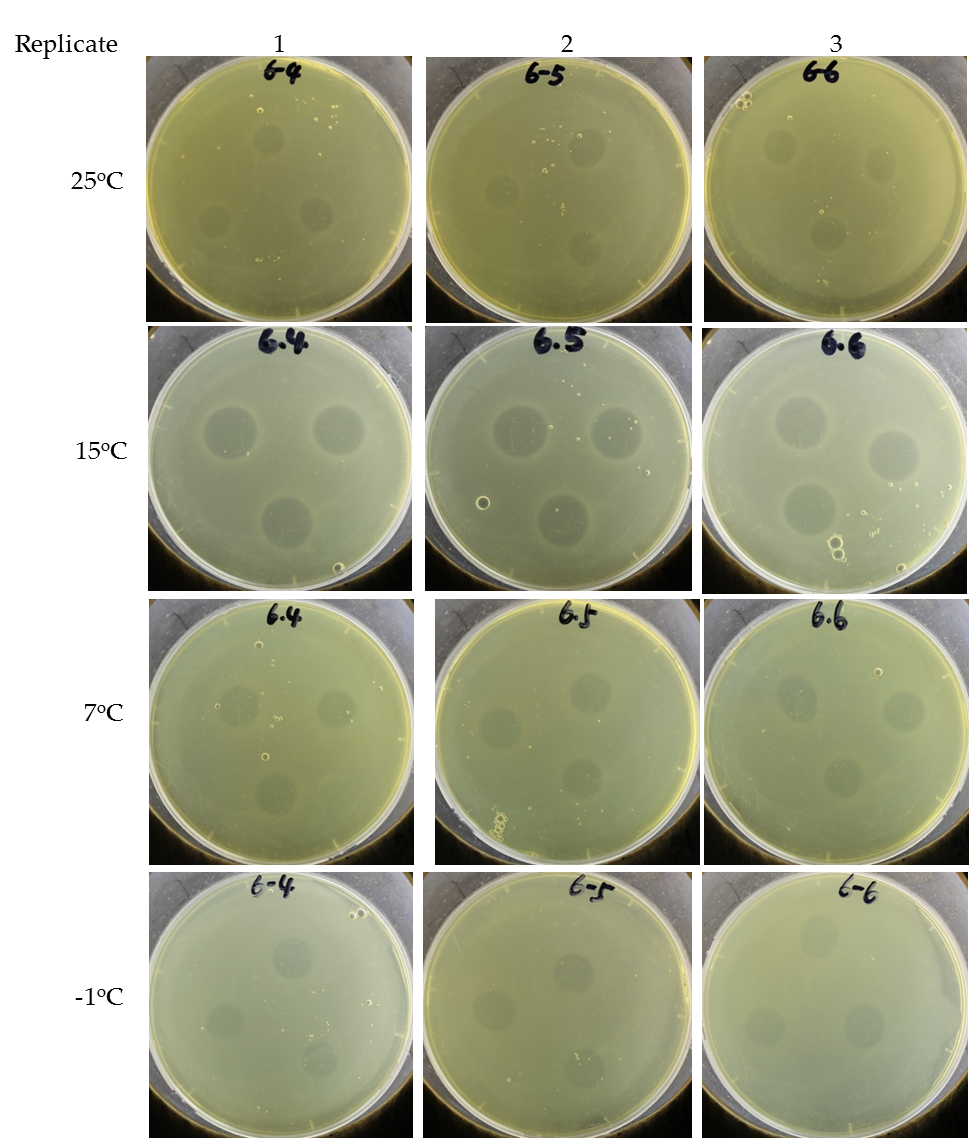


**Figure S4.** Representative pictures of inhibition zone showing the effect of temperature on D8c sensitivity (diameter of inhibition zone). Culture condition: BHI agar medium, 25 mM lactic acid, 0 mM glucose, pH 6.5, anaerobic.


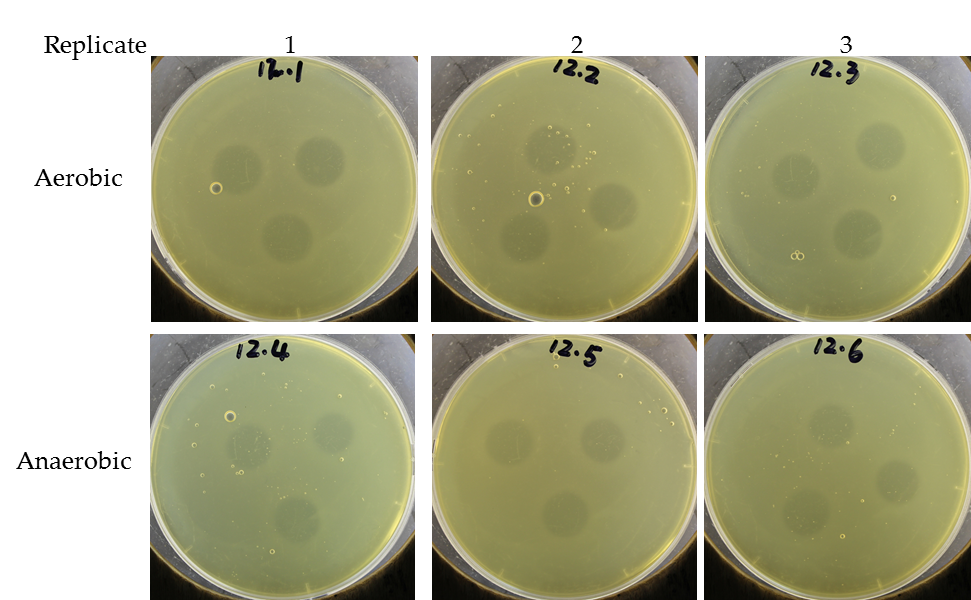


**Figure S5.** Representative pictures of inhibition zone showing the effect of atmosphere on D8c sensitivity (diameter of inhibition zone). Culture condition: BHI agar medium, 7^o^C, 0 mM lactic acid, 0.56 mM glucose, pH 6.5.


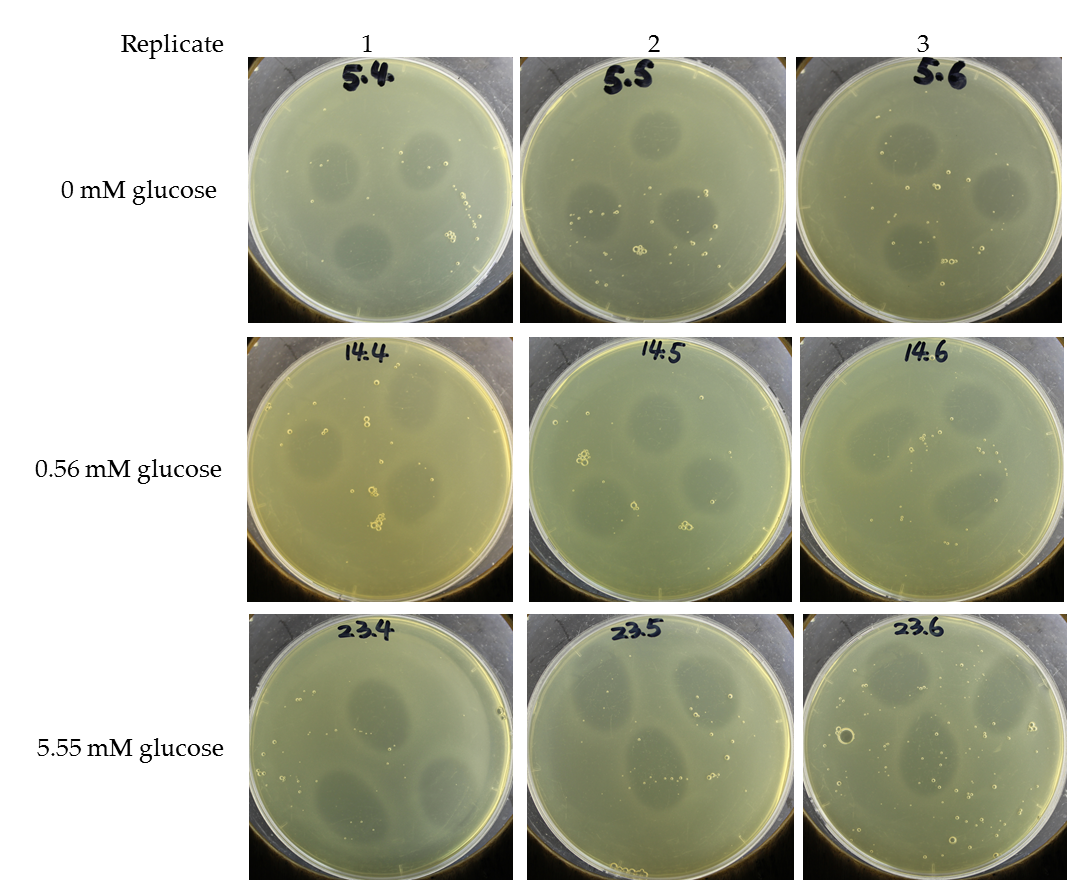


**Figure S6.** Representative pictures of inhibition zone showing the effect of glucose on D8c sensitivity (diameter of inhibition zone). Culture condition: BHI agar medium, 15^o^C, anaerobic, pH 6, 25 mM lactic acid.


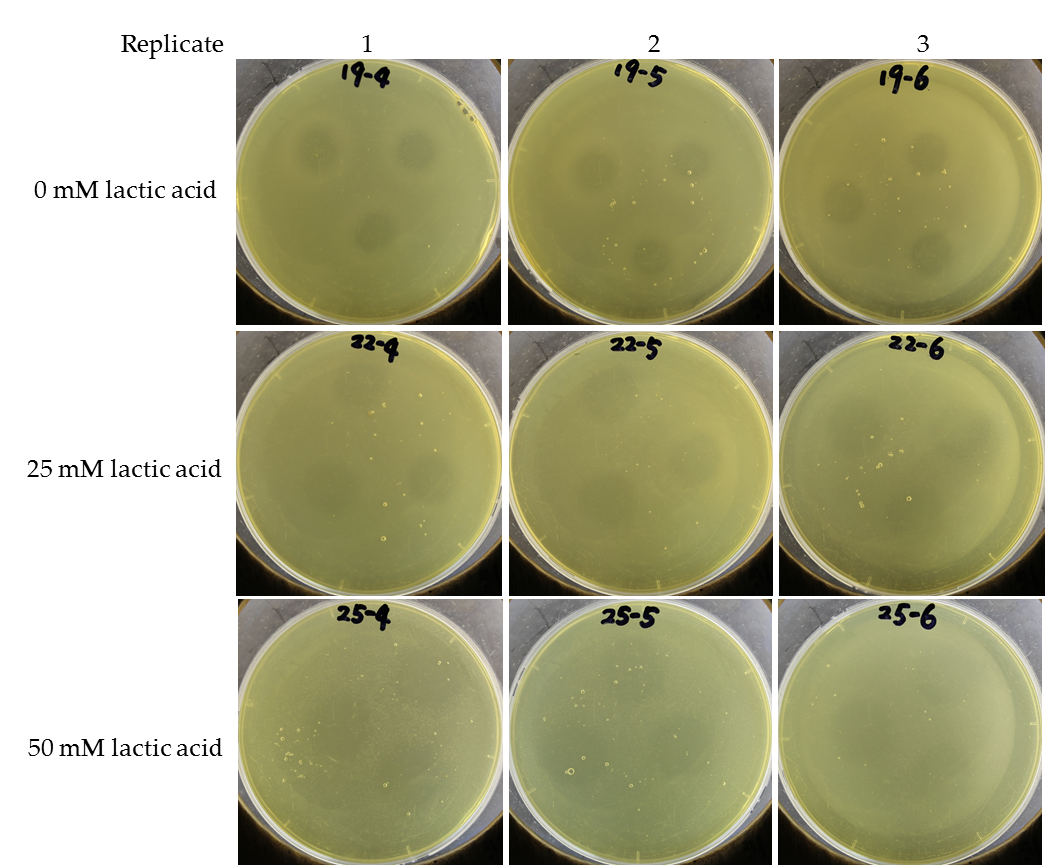


**Figure S7.** Representative pictures of inhibition zone showing the effect of lactic acid on D8c sensitivity (diameter of inhibition zone). Culture condition: BHI agar medium, 25^o^C, anaerobic, pH 5.5, 5.55 mM glucose.


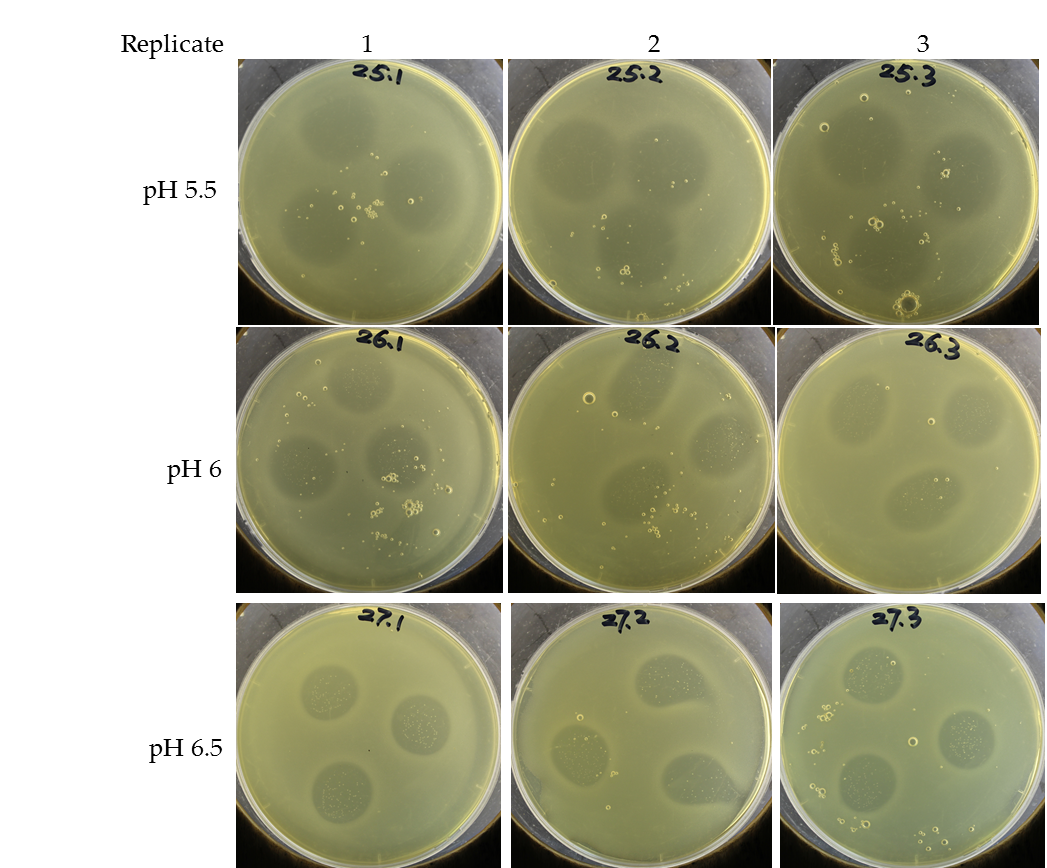


**Figure S8.** Representative pictures of inhibition zone showing the effect of pH on D8c sensitivity (diameter of inhibition zone). Culture condition: BHI agar medium, 15^o^C, aerobic, 50 mM lactic acid, 5.55 mM glucose.

**Figure S9.** Effect of pH, lactic acid and glucose on final cell density (FCD) in the study of *C. maltaromaticum* D8c sensitivity.

Error bars represent standard error of the mean. Means with the same letters are not significantly different. In this assay, a small piece (approximately 0.5 g) of agar was sampled from an area of mBHI agar not displaying an inhibition zone. Agar was weighed, pulverized, and then mixed with peptone water (bacteriological peptone 1 g/L, NaCl 8.5 g/L, pH 7.3 ± 0.2). CFU was determined by plate–count.

**Figure S10.** Effect of pH on growth rate of *C. maltaromaticum* D8c.

Culture condition: Brain heart infusion broth medium with pH 5.5, 6, and 6.5; 25^o^C. Error bars represent standard error of the mean. Means with the same letters are not significantly different.

**Figure S11.** Effect of incubation time on diameter of inhibition zone (DI) at different incubation days. Error bars represent standard deviation.

**Figure S12.** Effect of interactions between pH and glucose, and pH and lactic acid on diameter of inhibition zone (DI) at 25^o^C. Error bars are standard error of the mean. Means within the same level of pH were compared and different letters indicate significant differences.
