# Supplementary material for: Effect of Environmental Factors on Intra-Specific Inhibitory Activity of Carnobacterium maltaromaticum
Source: Microorganisms. 2017 Sep 14;5(3):59. doi: 10.3390/microorganisms5030059 (PMC5620650; doi:10.3390/microorganisms5030059)
Supplement: Supplementary file 1 [file microorganisms-05-00059-s001.zip › Supplementary file_Tables.docx]

Supplementary Materials: Effect of Environmental Factors on Intra-Specific Inhibitory Activity of *Carnobacterium altaromaticum*

Peipei Zhang, Mandeep Kaur, John P. Bowman, David A. Ratkowsky and Mark Tamplin

**Table S1.** Significance (*p*–value, F–test) of effect of environmental factors on production of inhibitory compounds by *C. maltaromaticum* D0h

|  | Source | Degrees of freedom | *p*–value (F–test) | |
| --- | --- | --- | --- | --- |
|  |  |  | Production per hour (AU/mL/h) | Production per CFU (logAU / logCFU) |
| ingle factor | Atmosphere | 1 | 0.2361 | 0.7306 |
|  | Glucose | 2 | 0.4043 | **0.0490** |
|  | LA | 2 | **0.0270** | 0.5875 |
|  | pH | 2 | **<0.0001** | **0.0028** |
| Two-factors interaction | Atmosphere*Glucose | 2 | 0.3050 | 0.9380 |
|  | Atmosphere*LA | 2 | 0.8899 | 0.0711 |
|  | Atmosphere*pH | 2 | 0.3708 | 0.4424 |
|  | Glucose*LA | 4 | 0.3262 | 0.5364 |
|  | Glucose*pH | 4 | 0.0017 | 0.3974 |
|  | LA*pH | 4 | **0.0098** | 0.6137 |

**Table S2.** D0h inhibitor production in various culture conditions

| **Medium No.** | **Glucose (mM)** | **Lactic acid (mM)** | **pH** | **Production per hour (AU/mL/h)** | | | | **Production rate (logAU/logCFU)** | |
| --- | --- | --- | --- | --- | --- | --- | --- | --- | --- |
|  |  |  |  | Aerobic | | Anaerobic | | Aerobic | **Anaerobic** |
|  |  |  |  | Replicate 1 | Replicate 2 | Replicate 1 | Replicate 2 |  |  |
| 1 | 0 | 0 | 5.5 | 64.79 | 74.58 | 49.30 | 130.67 | 1.04 | 0.71 |
| 2 | 0 | 0 | 6 | 155.03 | 86.05 | 91.04 | 78.40 | 1.61 | 2.06 |
| 3 | 0 | 0 | 6.5 | 60.40 | 66.60 | 73.20 | 70.60 | 0.99 | 1.38 |
| 4 | 0 | 25 | 5.5 | 16.66 | 27.66 | 9.61 | 24.86 | 1.23 | 1.58 |
| 5 | 0 | 25 | 6 | 21.74 | 110.14 | 22.13 | 76.88 | 1.80 | 1.11 |
| 6 | 0 | 25 | 6.5 | 73.09 | 99.05 | 78.30 | 191.13 | 0.88 | 1.31 |
| 7 | 0 | 50 | 5.5 | 49.72 | 58.87 | 0.00 | 0.00 | 0.78 | 0.00 |
| 8 | 0 | 50 | 6 | 110.78 | 131.21 | 106.20 | 119.57 | 1.62 | 1.51 |
| 9 | 0 | 50 | 6.5 | 95.97 | 257.83 | 72.59 | 232.54 | 1.17 | 1.30 |
| 10 | 0.56 | 0 | 5.5 | 44.42 | 55.71 | 56.50 | 43.83 | 0.35 | 2.63 |
| 11 | 0.56 | 0 | 6 | 125.74 | 101.60 | 101.19 | 94.58 | 1.36 | 1.41 |
| 12 | 0.56 | 0 | 6.5 | 76.38 | 76.41 | 78.02 | 64.86 | 1.12 | 1.54 |
| 13 | 0.56 | 25 | 5.5 | 66.67 | 65.15 | 17.58 | 32.26 | 1.66 | 0.49 |
| 14 | 0.56 | 25 | 6 | 111.73 | 70.09 | 82.85 | 49.77 | 1.57 | 1.17 |
| 15 | 0.56 | 25 | 6.5 | 124.31 | 59.08 | 110.23 | 71.96 | 1.15 | 1.75 |
| 16 | 0.56 | 50 | 5.5 | 75.38 | 53.10 | 0.00 | 0.00 | 1.41 | 0.00 |
| 17 | 0.56 | 50 | 6 | 43.93 | 203.55 | 30.28 | 120.07 | 1.78 | 0.83 |
| 18 | 0.56 | 50 | 6.5 | 83.03 | 85.82 | 75.68 | 71.98 | 0.96 | 1.50 |
| 19 | 5.55 | 0 | 5.5 | 36.20 | 46.48 | 0.00 | 0.00 | 0.67 | 0.00 |
| 20 | 5.55 | 0 | 6 | 86.78 | 90.22 | 76.71 | 109.72 | 0.99 | 1.38 |
| 21 | 5.55 | 0 | 6.5 | 107.00 | 186.62 | 140.71 | 74.82 | 1.07 | 1.00 |
| 22 | 5.55 | 25 | 5.5 | 0.00 | 0.00 | 0.00 | 0.00 | 0.00 | 0.00 |
| 23 | 5.55 | 25 | 6 | 74.89 | 69.65 | 93.67 | 139.02 | 1.03 | 1.08 |
| 24 | 5.55 | 25 | 6.5 | 95.35 | 164.30 | 79.74 | 102.06 | 1.37 | 0.60 |
| 25 | 5.55 | 50 | 5.5 | 25.79 | 23.55 | 0.00 | 0.00 | 0.95 | 0.00 |
| 26 | 5.55 | 50 | 6 | 45.49 | 90.45 | 137.34 | 159.15 | 0.88 | 1.41 |
| 27 | 5.55 | 50 | 6.5 | 133.64 | 190.15 | 206.42 | 229.52 | 1.51 | 1.43 |

**Table S3.** Significance (*p–*value, F–test) of effect of environmental factors on *C. maltaromaticum* D8c sensitivity to *C. maltaromaticum* D0h CFS

|  | **Source** | **Degrees of freedom** | ***P* value (F-test)** |
| --- | --- | --- | --- |
| Single factor | Temperature | 3 | **<0.0001** |
|  | Atmosphere | 1 | **<0.0001** |
|  | Glucose | 2 | **<0.0001** |
|  | LA | 2 | **<0.0001** |
|  | pH | 2 | **<0.0001** |
| Two-factors interaction | Temerature*Atmosphere | 3 | **<0.0001** |
|  | Temperature*Glucose | 6 | **<0.0001** |
|  | Temperature*LA | 6 | **<0.0001** |
|  | Temperature*pH | 6 | **0.0003** |
|  | Atmosphere*Glucose | 2 | 0.0545 |
|  | Atmosphere*LA | 2 | 0.6659 |
|  | Atmosphere*pH | 2 | 0.5249 |
|  | Glucose*LA | 4 | 0.3396 |
|  | Glucose*pH | 4 | **<0.0001** |
|  | LA*pH | 4 | **<0.0001** |

**Table S4.** The sensitivity of *C. maltaromaticum* (diameter of inhibition zone, DI) D8c to *C. maltaromaticum* D0h inhibition under aerobic conditions

| **Medium no.** | **T (^o^C)** | **Glucose (mM)** | **pH** | **LA (mM)** | **DI (mm)** |
| --- | --- | --- | --- | --- | --- |
| 1 | -1 | 0 | 5.5 | 0 | 13.93 |
| 2 | -1 | 0 | 6 | 0 | 13.27 |
| 3 | -1 | 0 | 6.5 | 0 | 13.86 |
| 4 | -1 | 0 | 5.5 | 25 | . |
| 5 | -1 | 0 | 6 | 25 | 15 |
| 6 | -1 | 0 | 6.5 | 25 | 13.63 |
| 7 | -1 | 0 | 5.5 | 50 | . |
| 8 | -1 | 0 | 6 | 50 | 14.56 |
| 9 | -1 | 0 | 6.5 | 50 | 14.34 |
| 10 | -1 | 0.56 | 5.5 | 0 | 13.84 |
| 11 | -1 | 0.56 | 6 | 0 | 12.72 |
| 12 | -1 | 0.56 | 6.5 | 0 | 13.08 |
| 13 | -1 | 0.56 | 5.5 | 25 | 15.46 |
| 14 | -1 | 0.56 | 6 | 25 | 15.58 |
| 15 | -1 | 0.56 | 6.5 | 25 | 15.31 |
| 16 | -1 | 0.56 | 5.5 | 50 | . |
| 17 | -1 | 0.56 | 6 | 50 | 15.16 |
| 18 | -1 | 0.56 | 6.5 | 50 | 14.61 |
| 19 | -1 | 5.55 | 5.5 | 0 | 14.96 |
| 20 | -1 | 5.55 | 6 | 0 | 14.99 |
| 21 | -1 | 5.55 | 6.5 | 0 | 14.25 |
| 22 | -1 | 5.55 | 5.5 | 25 | . |
| 23 | -1 | 5.55 | 6 | 25 | 15.21 |
| 24 | -1 | 5.55 | 6.5 | 25 | 13.92 |
| 25 | -1 | 5.55 | 5.5 | 50 | . |
| 26 | -1 | 5.55 | 6 | 50 | 17.88 |
| 27 | -1 | 5.55 | 6.5 | 50 | 15.43 |
| 1 | 7 | 0 | 5.5 | 0 | 13.76 |
| 2 | 7 | 0 | 6 | 0 | 15.9 |
| 3 | 7 | 0 | 6.5 | 0 | 16.68 |
| 4 | 7 | 0 | 5.5 | 25 | 16.4 |
| 5 | 7 | 0 | 6 | 25 | 16.76 |
| 6 | 7 | 0 | 6.5 | 25 | 16.03 |
| 7 | 7 | 0 | 5.5 | 50 | 17.93 |
| 8 | 7 | 0 | 6 | 50 | 14.78 |
| 9 | 7 | 0 | 6.5 | 50 | 14.46 |
| 10 | 7 | 0.56 | 5.5 | 0 | 18.07 |
| 11 | 7 | 0.56 | 6 | 0 | 17.2 |
| 12 | 7 | 0.56 | 6.5 | 0 | 16.78 |
| 13 | 7 | 0.56 | 5.5 | 25 | 15.31 |
| 14 | 7 | 0.56 | 6 | 25 | 17 |
| 15 | 7 | 0.56 | 6.5 | 25 | 16.49 |
| 16 | 7 | 0.56 | 5.5 | 50 | 13.15 |
| 17 | 7 | 0.56 | 6 | 50 | 16.01 |
| 18 | 7 | 0.56 | 6.5 | 50 | 14.8 |
| 19 | 7 | 5.55 | 5.5 | 0 | 19.92 |
| 20 | 7 | 5.55 | 6 | 0 | 19.12 |
| 21 | 7 | 5.55 | 6.5 | 0 | 15.85 |
| 22 | 7 | 5.55 | 5.5 | 25 | 17.99 |
| 23 | 7 | 5.55 | 6 | 25 | 18.59 |
| 24 | 7 | 5.55 | 6.5 | 25 | 17.53 |
| 25 | 7 | 5.55 | 5.5 | 50 | 22.51 |
| 26 | 7 | 5.55 | 6 | 50 | 17.06 |
| 27 | 7 | 5.55 | 6.5 | 50 | 17.48 |
| 1 | 15 | 0 | 5.5 | 0 | 18.22 |
| 2 | 15 | 0 | 6 | 0 | 18.03 |
| 3 | 15 | 0 | 6.5 | 0 | 16.62 |
| 4 | 15 | 0 | 5.5 | 25 | 20.34 |
| 5 | 15 | 0 | 6 | 25 | 18.84 |
| 6 | 15 | 0 | 6.5 | 25 | 17.26 |
| 7 | 15 | 0 | 5.5 | 50 | 19.52 |
| 8 | 15 | 0 | 6 | 50 | 18.25 |
| 9 | 15 | 0 | 6.5 | 50 | 17.32 |
| 10 | 15 | 0.56 | 5.5 | 0 | 19.98 |
| 11 | 15 | 0.56 | 6 | 0 | 17.87 |
| 12 | 15 | 0.56 | 6.5 | 0 | 19.47 |
| 13 | 15 | 0.56 | 5.5 | 25 | 21.57 |
| 14 | 15 | 0.56 | 6 | 25 | 19.48 |
| 15 | 15 | 0.56 | 6.5 | 25 | 18.99 |
| 16 | 15 | 0.56 | 5.5 | 50 | 25.73 |
| 17 | 15 | 0.56 | 6 | 50 | 19.88 |
| 18 | 15 | 0.56 | 6.5 | 50 | 18.37 |
| 19 | 15 | 5.55 | 5.5 | 0 | 20.86 |
| 20 | 15 | 5.55 | 6 | 0 | 20.59 |
| 21 | 15 | 5.55 | 6.5 | 0 | 22.05 |
| 22 | 15 | 5.55 | 5.5 | 25 | 23.21 |
| 23 | 15 | 5.55 | 6 | 25 | 22 |
| 24 | 15 | 5.55 | 6.5 | 25 | 19.82 |
| 25 | 15 | 5.55 | 5.5 | 50 | 27.36 |
| 26 | 15 | 5.55 | 6 | 50 | 22.16 |
| 27 | 15 | 5.55 | 6.5 | 50 | 19.9 |
| 1 | 25 | 0 | 5.5 | 0 | 10.91 |
| 2 | 25 | 0 | 6 | 0 | 12.44 |
| 3 | 25 | 0 | 6.5 | 0 | 11.77 |
| 4 | 25 | 0 | 5.5 | 25 | 14.03 |
| 5 | 25 | 0 | 6 | 25 | 12.38 |
| 6 | 25 | 0 | 6.5 | 25 | 12.27 |
| 7 | 25 | 0 | 5.5 | 50 | 15.02 |
| 8 | 25 | 0 | 6 | 50 | 12.06 |
| 9 | 25 | 0 | 6.5 | 50 | 12.67 |
| 10 | 25 | 0.56 | 5.5 | 0 | 12.45 |
| 11 | 25 | 0.56 | 6 | 0 | 13.63 |
| 12 | 25 | 0.56 | 6.5 | 0 | 12.62 |
| 13 | 25 | 0.56 | 5.5 | 25 | 12.71 |
| 14 | 25 | 0.56 | 6 | 25 | 13.54 |
| 15 | 25 | 0.56 | 6.5 | 25 | 13.53 |
| 16 | 25 | 0.56 | 5.5 | 50 | 13.53 |
| 17 | 25 | 0.56 | 6 | 50 | 16.75 |
| 18 | 25 | 0.56 | 6.5 | 50 | 14.57 |
| 19 | 25 | 5.55 | 5.5 | 0 | 14.08 |
| 20 | 25 | 5.55 | 6 | 0 | 13.43 |
| 21 | 25 | 5.55 | 6.5 | 0 | 14.5 |
| 22 | 25 | 5.55 | 5.5 | 25 | 20.19 |
| 23 | 25 | 5.55 | 6 | 25 | 14.96 |
| 24 | 25 | 5.55 | 6.5 | 25 | 14.39 |
| 25 | 25 | 5.55 | 5.5 | 50 | 19.68 |
| 26 | 25 | 5.55 | 6 | 50 | 16.01 |
| 27 | 25 | 5.55 | 6.5 | 50 | 13.6 |

Note: ‘.’, missing data.

**Table S5.** The sensitivity of *C. maltaromaticum* (diameter of inhibition zone, DI) D8c to *C. maltaromaticum* D0h inhibition under anaerobic conditions

| **Medium no.** | **T (^o^C)** | **Glucose (mM)** | **pH** | **LA (mM)** | **DI (mm)** |
| --- | --- | --- | --- | --- | --- |
| 1 | -1 | 0 | 5.5 | 0 | 16.39 |
| 2 | -1 | 0 | 6 | 0 | 13.89 |
| 3 | -1 | 0 | 6.5 | 0 | 13.94 |
| 4 | -1 | 0 | 5.5 | 25 | 13.19 |
| 5 | -1 | 0 | 6 | 25 | 13.77 |
| 6 | -1 | 0 | 6.5 | 25 | 13.36 |
| 7 | -1 | 0 | 5.5 | 50 | . |
| 8 | -1 | 0 | 6 | 50 | 13.86 |
| 9 | -1 | 0 | 6.5 | 50 | 14.81 |
| 10 | -1 | 0.56 | 5.5 | 0 | 13.43 |
| 11 | -1 | 0.56 | 6 | 0 | 13.93 |
| 12 | -1 | 0.56 | 6.5 | 0 | 13.37 |
| 13 | -1 | 0.56 | 5.5 | 25 | 12.37 |
| 14 | -1 | 0.56 | 6 | 25 | 14.43 |
| 15 | -1 | 0.56 | 6.5 | 25 | 14.24 |
| 16 | -1 | 0.56 | 5.5 | 50 | 14.35 |
| 17 | -1 | 0.56 | 6 | 50 | 14.01 |
| 18 | -1 | 0.56 | 6.5 | 50 | 13.87 |
| 19 | -1 | 5.55 | 5.5 | 0 | 15.15 |
| 20 | -1 | 5.55 | 6 | 0 | 12.63 |
| 21 | -1 | 5.55 | 6.5 | 0 | 14.77 |
| 22 | -1 | 5.55 | 5.5 | 25 | 18.09 |
| 23 | -1 | 5.55 | 6 | 25 | 13.92 |
| 24 | -1 | 5.55 | 6.5 | 25 | 13.56 |
| 25 | -1 | 5.55 | 5.5 | 50 | . |
| 26 | -1 | 5.55 | 6 | 50 | 14.15 |
| 27 | -1 | 5.55 | 6.5 | 50 | 14.72 |
| 1 | 7 | 0 | 5.5 | 0 | 13.57 |
| 2 | 7 | 0 | 6 | 0 | 14.27 |
| 3 | 7 | 0 | 6.5 | 0 | 16.91 |
| 4 | 7 | 0 | 5.5 | 25 | 13.42 |
| 5 | 7 | 0 | 6 | 25 | 16.51 |
| 6 | 7 | 0 | 6.5 | 25 | 13.9 |
| 7 | 7 | 0 | 5.5 | 50 | 15.12 |
| 8 | 7 | 0 | 6 | 50 | 12.92 |
| 9 | 7 | 0 | 6.5 | 50 | 14.22 |
| 10 | 7 | 0.56 | 5.5 | 0 | 14.82 |
| 11 | 7 | 0.56 | 6 | 0 | 15.16 |
| 12 | 7 | 0.56 | 6.5 | 0 | 15.33 |
| 13 | 7 | 0.56 | 5.5 | 25 | 13.2 |
| 14 | 7 | 0.56 | 6 | 25 | 15.25 |
| 15 | 7 | 0.56 | 6.5 | 25 | 13.52 |
| 16 | 7 | 0.56 | 5.5 | 50 | 14.05 |
| 17 | 7 | 0.56 | 6 | 50 | 14.78 |
| 18 | 7 | 0.56 | 6.5 | 50 | 13.69 |
| 19 | 7 | 5.55 | 5.5 | 0 | 14.22 |
| 20 | 7 | 5.55 | 6 | 0 | 15.62 |
| 21 | 7 | 5.55 | 6.5 | 0 | 13.4 |
| 22 | 7 | 5.55 | 5.5 | 25 | 14.17 |
| 23 | 7 | 5.55 | 6 | 25 | 14.95 |
| 24 | 7 | 5.55 | 6.5 | 25 | 15.37 |
| 25 | 7 | 5.55 | 5.5 | 50 | 16.09 |
| 26 | 7 | 5.55 | 6 | 50 | 14.48 |
| 27 | 7 | 5.55 | 6.5 | 50 | 14.92 |
| 1 | 15 | 0 | 5.5 | 0 | 18 |
| 2 | 15 | 0 | 6 | 0 | 17.53 |
| 3 | 15 | 0 | 6.5 | 0 | 17.47 |
| 4 | 15 | 0 | 5.5 | 25 | 19.05 |
| 5 | 15 | 0 | 6 | 25 | 18.67 |
| 6 | 15 | 0 | 6.5 | 25 | 17.27 |
| 7 | 15 | 0 | 5.5 | 50 | 22.87 |
| 8 | 15 | 0 | 6 | 50 | 18.62 |
| 9 | 15 | 0 | 6.5 | 50 | 17.81 |
| 10 | 15 | 0.56 | 5.5 | 0 | 18.5 |
| 11 | 15 | 0.56 | 6 | 0 | 18.01 |
| 12 | 15 | 0.56 | 6.5 | 0 | 18.47 |
| 13 | 15 | 0.56 | 5.5 | 25 | 21.78 |
| 14 | 15 | 0.56 | 6 | 25 | 20.13 |
| 15 | 15 | 0.56 | 6.5 | 25 | 19.31 |
| 16 | 15 | 0.56 | 5.5 | 50 | 22.8 |
| 17 | 15 | 0.56 | 6 | 50 | 20.01 |
| 18 | 15 | 0.56 | 6.5 | 50 | 18.83 |
| 19 | 15 | 5.55 | 5.5 | 0 | 23.03 |
| 20 | 15 | 5.55 | 6 | 0 | 20.59 |
| 21 | 15 | 5.55 | 6.5 | 0 | 20.99 |
| 22 | 15 | 5.55 | 5.5 | 25 | 23.7 |
| 23 | 15 | 5.55 | 6 | 25 | 22.91 |
| 24 | 15 | 5.55 | 6.5 | 25 | 19.95 |
| 25 | 15 | 5.55 | 5.5 | 50 | 24.11 |
| 26 | 15 | 5.55 | 6 | 50 | 22.19 |
| 27 | 15 | 5.55 | 6.5 | 50 | 20.43 |
| 1 | 25 | 0 | 5.5 | 0 | 10.34 |
| 2 | 25 | 0 | 6 | 0 | 11.88 |
| 3 | 25 | 0 | 6.5 | 0 | 11.75 |
| 4 | 25 | 0 | 5.5 | 25 | 14.03 |
| 5 | 25 | 0 | 6 | 25 | 12.01 |
| 6 | 25 | 0 | 6.5 | 25 | 11.33 |
| 7 | 25 | 0 | 5.5 | 50 | 15.81 |
| 8 | 25 | 0 | 6 | 50 | 12.3 |
| 9 | 25 | 0 | 6.5 | 50 | 12.35 |
| 10 | 25 | 0.56 | 5.5 | 0 | 11.99 |
| 11 | 25 | 0.56 | 6 | 0 | 12.1 |
| 12 | 25 | 0.56 | 6.5 | 0 | 12.48 |
| 13 | 25 | 0.56 | 5.5 | 25 | 12.31 |
| 14 | 25 | 0.56 | 6 | 25 | 13.5 |
| 15 | 25 | 0.56 | 6.5 | 25 | 12.83 |
| 16 | 25 | 0.56 | 5.5 | 50 | 16.3 |
| 17 | 25 | 0.56 | 6 | 50 | 14.63 |
| 18 | 25 | 0.56 | 6.5 | 50 | 13.1 |
| 19 | 25 | 5.55 | 5.5 | 0 | 13.19 |
| 20 | 25 | 5.55 | 6 | 0 | 13.26 |
| 21 | 25 | 5.55 | 6.5 | 0 | 13.56 |
| 22 | 25 | 5.55 | 5.5 | 25 | 18.32 |
| 23 | 25 | 5.55 | 6 | 25 | 15.31 |
| 24 | 25 | 5.55 | 6.5 | 25 | 13.52 |
| 25 | 25 | 5.55 | 5.5 | 50 | 20.47 |
| 26 | 25 | 5.55 | 6 | 50 | 16.07 |
| 27 | 25 | 5.55 | 6.5 | 50 | 14.2 |

Note: ‘.’, missing data.

**Table S6.** Significance (*p*–value*,* F–test) of effects of environmental factors on *C. maltaromaticum* D8c sensitivity to *C. maltaromaticum* D0h CFS at 25^o^C

|  | **Source** | **Degrees of freedom** | ***P* value (F-test)** |
| --- | --- | --- | --- |
| Single factor | Atmosphere | 1 | 0.2164 |
|  | Glucose | 2 | **< 0.0001** |
|  | LA | 2 | **< 0.0001** |
|  | pH | 2 | **< 0.0001** |
| Two-factors interaction | Atmosphere*Glucose | 2 | 0.9186 |
|  | Atmosphere*LA | 2 | 0.4370 |
|  | Atmosphere*pH | 2 | 0.6412 |
|  | Glucose*LA | 4 | 0.1343 |
|  | Glucose*pH | 4 | **0.0002** |
|  | LA*pH | 4 | **0.0003** |
